# Regional shifts in production and trade in the metal markets: a comparison of China, the EU, and the US

**DOI:** 10.1007/s13563-022-00321-7

**Published:** 2022-05-11

**Authors:** Johannes Perger

**Affiliations:** German Mineral Resources Agency, Federal Institute for Geosciences and Natural Resources, Hanover, Germany

**Keywords:** Base metals, Processing, Trade, Asia, Europe, North America

## Abstract

Metal raw materials are essential for industrial production. Global metal markets have changed considerably since the start of the twenty-first century, China being the most influential driver of this change. With China’s economic rise, metal demand has skyrocketed, and global production shares in metal raw materials and trade volumes have shifted towards the People’s Republic. While China has become the dominant location for global metal production and consumption, older industrial economies like the European countries or the US have lost global share. This paper aims to compare the different developments in China, the European Union and the US regarding markets for the base metals aluminium, copper, lead, nickel, tin and zinc as well as iron and steel. The analysis covers movements in industrial production, metal production locations (mining – refined production – refined consumption) and metal trade volumes (ores and concentrates – waste and scrap – refined products and articles thereof).

## Introduction

China’s rise to become a major economic power over the past decades has been rapid. The economic upswing in the People’s Republic (PRC) was achieved through favourable production conditions, good integration into global value chains, strategic partnerships and export-oriented industrialisation controlled by state leadership. For many foreign companies, China was also attractive because of its size as a labour and consumer market. In 2018, China had a population of 1430 million (18.7% of the world’s population), compared to 510 million (6.7%) in the European Union (EU) and 330 million (4.3%) in the United States (US) (UN/WPP 2019). With increasing globalisation, many industries from Europe and the US have moved to more favourable production locations in terms of lower production costs, weaker social and environmental legislation, and proximity to growing target markets. The most important destination has been, and probably still is, China. But how are these developments and shifts reflected in the commodity markets? Have extraction and production sites for metal raw materials within the three economic powers actually been relocated? How have commodity trade flows involving China, the EU and the US changed?

The aim of this study is to compare the positions of China, EU and US in the metal markets. It uses a range of economic and commodity indicators to present their respective positions in a data-driven analysis. In order to make long-term developments visible, indicators are always considered for the reference years 2002 and 2018. These two years were selected for the following reasons: (1) Some of the most recent commodity data refer to 2018, when the UK was still part of the European Union in all datasets. (2) Trade data from the Global Trade Atlas (GTA 2020) are available for the EU’s external commodity trade from 2002, which is also the year China joined the World Trade Organisation (WTO). For better comparison, the EU is defined as the EU-28 in both years, which includes the UK as well as especially Eastern European countries that actually joined the economic area in 2004, 2007 and 2013. The commodities considered are the base metals aluminium, lead, copper, nickel, zinc and tin as well as iron and steel.

## Industrial production and GDP

China has experienced an enormous economic boom over the past 30 years. Ranking eleventh among the largest economies by GDP in 1990, the PRC had risen to second place in 2018. The US are still ahead in this ranking, but their lead is dwindling. If the EU-28 (including the UK) are grouped together as one economic unit, the EU can be considered as the third big economic power (World Bank 2020, calculations in “constant 2010 US$”).

**Fig. 1:**
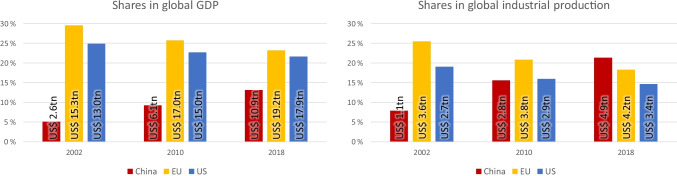
Shares of China, EU and US in global GDP and industrial production (World Bank 2020)

In 2002, China’s share in global economic output was 5.1%, the EU had a share of 29.5% and the US a share of 25% (Fig. 1). Thus, the EU and the US together accounted for more than half of global economic output. Since then, the Chinese economy has grown at around 9.7% per year, while the economies of the EU and the US have grown by only 1.2% and 1.7% per year, respectively. This has significantly shifted the balance within the global economy towards China (World Bank 2020, calculations in “constant 2010 US$” and with best-fit growth [calculated via logarithmic calculus of the values and linear regression]). In 2018, China accounted for 13.1%, the EU for 23.3% and the US for 21.6%—the EU and the US together still generating 45% of global GDP (Fig. 1). Thus, in terms of total output across all economic sectors (agriculture, industry, services), the Western states continue to hold the top positions.

The situation is different for industrial production, which is dependent on raw materials. In 2002, total output in the industrial sector in the EU and the US respectively was more than twice as high as in China. China accounted for 8% of global industrial production, the EU for 25.6% and the US for 19.1% (Fig. 1). Since then, industrial production in China has grown by about 10.4% per year, while the EU and the US have remained at growth rates of about half a percent. By 2018, China’s industrial production had thus more than quadrupled, overtaking both the EU and the US (World Bank 2020, calculations in “constant 2010 US$” and with best-fit growth). In 2018, China accounted for about 21.4% of global industrial production, the EU for 18.1% and the US for 14.2%—an indication of China’s continued position as the “workshop of the world”.

Just over 40% of China’s GDP was generated in the industrial sector in 2018 (US$ 4.9 trillion of US$ 10.9 trillion)—down from around 45% in 2002 and 2010. In the EU, this share was just over 20%, in the US just under 20%. This underscores the great importance that the raw material-intensive industrial sector has for the Chinese economy (World Bank 2020).

Overall, the indicators show the crucial importance of China’s industrial production for the Chinese economy and, in the past 20 years, also for the global economy. The EU and the US have lost in importance in terms of their share in global industrial production. The following chapter shows that the shifts in industrial production have also changed the locations of raw material production.

## Mining, refined production and refined consumption

The production of industrial goods requires a large amount of metal raw materials. Both supply-driven effects (use of new deposits) and demand-driven effects (relocation of industrial production) have led to a relocation of raw material production and raw material trade flows since the beginning of the century. In this study, metal raw materials are classified according to their processing stages into mine production, refined production and refined consumption.

Overall, Table 1 illustrates the dominance of China as a location for raw material production in recent decades. In 2018, China accounted for very high shares of global refined metal production and consumption for all metals under review, while the EU and the US had significantly lower shares than they did at the beginning of the millennium. But the three major economic powers had similar levels of industrial production. This shows that much of China’s industrial production is still closer to the beginning of the value chain, mostly in the production of primary products and intermediates. Today, however, China’s strength lies no longer in the raw materials sector only, but equally in the manufacture of high-quality products. Value creation in the EU and the US, on the other hand, focuses in general on higher processing stages.

**Table 1:** Global shares of China, EU and US in mining, refined production and refined consumption of metals in 2002 and 2018, as well as the changes of the absolute amounts in factors in the time interval. If at least one of the shares of 2002 or 2018 is rounded to 0.0%, the factor is not calculated. Refined production for iron/steel constitutes raw steel production (BGR 2021)^1^

		Mining			Refined production			Refined consumption		
		2002	2018	Factor	2002	2018	Factor	2002	2018	Factor
Aluminium	China	8.8%	19.0%	4.8	16.8%	57.7%	8.1	16.1%	55.3%	8.1
	EU	2.3%	0.0%	-	11.1%	3.2%	0.9	25.1%	12.5%	1.2
	US	0.0%	0.0%	-	10.4%	1.4%	0.3	21.6%	7.7%	0.8
Lead	China	22.1%	44.8%	3.3	19.4%	42.2%	3.7	14.0%	40.9%	5.1
	EU	7.6%	3.9%	0.8	24.6%	14.9%	1.0	27.7%	15.3%	1.0
	US	15.6%	6.0%	0.6	19.9%	9.8%	0.8	22.4%	9.4%	0.7
Copper	China	4.3%	7.8%	2.8	10.5%	38.5%	5.8	18.5%	51.0%	4.5
	EU	5.7%	4.4%	1.2	15.9%	11.2%	1.1	26.5%	13.0%	0.8
	US	8.4%	5.9%	1.1	9.8%	4.6%	0.7	15.5%	7.4%	0.8
Nickel	China	4.5%	4.3%	1.8	4.5%	31.0%	12.8	7.6%	51.6%	12.8
	EU	2.1%	2.7%	2.7	10.2%	5.4%	1.1	38.0%	14.0%	0.7
	US	0.0%	0.8%	-	0.0%	0.0%	-	10.1%	6.0%	1.2
Iron/steel	China	11.7%	15.8%	3.1	20.1%	51.1%	5.1	23.3%	48.8%	4.4
	EU	2.5%	1.3%	1.2	20.7%	9.2%	0.9	19.5%	10.0%	1.1
	US	5.3%	2.3%	1.0	10.1%	4.8%	0.9	13.1%	5.8%	0.9
Zinc	China	18.1%	33.2%	2.7	21.8%	43.1%	2.6	18.5%	47.4%	3.7
	EU	8.4%	5.5%	0.9	25.1%	15.8%	0.8	26.3%	15.2%	0.8
	US	8.8%	6.6%	1.1	3.7%	0.9%	0.3	14.1%	6.3%	0.7
Tin	China	31.8%	29.8%	1.2	30.6%	47.7%	2.0	22.5%	45.1%	2.5
	EU	0.0%	0.0%	-	3.4%	3.8%	1.4	21.4%	16.5%	1.0
	US	0.0%	0.0%	-	0.0%	0.0%	-	15.8%	8.7%	0.7
Avg. shares	China	14.5%	14.5%	-	17.7%	44.5%	-	17.2%	49.0%	-
	EU	4.1%	2.6%	-	15.9%	9.1%	-	26.4%	13.8%	-
	US	5.5%	3.2%	-	7.7%	3.1%	-	16.1%	8.0%	-
	∑	24.1%	20.3%	-	41.3%	56.7%	-	59.7%	70.8%	-

^1^A factor of “1.0” indicates that the same amount of a commodity was mined/produced/consumed in 2018 as in 2002. A factor of “2.0” means that the quantity has doubled in the time interval

In order to interpret the results correctly, the development in the absolute volumes of raw materials must also be taken into account. Just because a country’s global share is reduced, the absolute quantities of raw materials mined, produced or consumed in that country do not necessarily decline as well. The “Factor” columns in Table 1 show changes in domestic mining, refined production and refined consumption between 2002 and 2018 for China, the EU and the US, based on factors. In China, refined production and consumption of all metals under review multiplied within the time interval, nickel and aluminium being the metals with the highest growth rates. In the EU, refined production and consumption generally maintained their 2002 levels (most factors close to 1.0), while tin-refined production increased the most and nickel-refined consumption dropped the most. In the US, refined production and consumption in general dropped sharply (just nickel consumption grew).

Overall, the indicators show that China has become the most important location for refined production and consumption in the commodity markets reviewed. Its high global shares in the processing stages and the multiplication of processed raw material volumes illustrate this. However, neither China nor the EU or the US can meet their total metal raw material demand from domestic mining operations. The required trade volumes are shown in the next chapter.

## Foreign trade

**Fig. 2:**
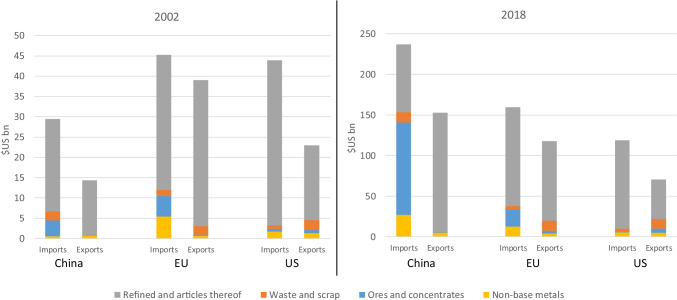
Foreign trade in metals for China, EU and US in 2002 and 2018 (GTA 2020)

China, the EU und the US are major players in the international trade in metal raw materials. Given that their shares in raw materials production are lower than those in refined consumption, the three economic powers are especially dependent on imports of metal pre-products. Figure 2 shows the value of foreign trade in metal ores and concentrates, waste and scrap, as well as refined products and articles thereof (refined products, semi-finished products and simple products such as nails, rods, wires, sheets and strips) for China, the EU and the US in 2002 and 2018.^1^

In 2002, the EU was both the largest importer and exporter of the commodity trade groups considered, with the US ranking second and China third. By 2018, this order had changed completely. China had become the largest importer and exporter of metal commodities, followed by the EU and the US. The trade of all three economic powers is dominated by imports; i.e. they are net importers of metal raw materials (see Fig. 2).

In 2002, ores and concentrates of the metals under consideration accounted for 13% of China’s metal imports, scrap and waste for 8%, and products for 77%. These ratios had shifted significantly by 2018. In 2018, China’s metal imports comprised 48% ores and concentrates of the commodities under consideration, 5% scrap and waste, and only 35% products. This shows that production in China has become significantly more vertically integrated between 2002 and 2018 to include all stages in the production chain from ore to higher products within the country. Imports arrived in China from the all over the world. In both years, China’s metal exports contained very few ores and concentrates or waste and scrap, and exports therefore mainly included refined products and goods made from them. Significant portions of sales went to Asia and the US (see Appendix, Fig. 4).

Shares of EU metal imports hardly changed at all between the two reference years; ores and concentrates of the metals under consideration accounted for around 12% in both years, waste and scrap for 3% and products for 75%. Ores and concentrates came mainly from American countries. The share of ores and concentrates in EU metal exports grew from 1 to 3% and of wastes and scrap from 5 to 10%, while the share of products in exports declined. A large share of ores and concentrates and of waste and scrap went to Asia (see Appendix, Fig. 6).

In both years under review, most metal imports into the US were of refined raw materials and articles thereof (shares of over 90% in each case). Imports of ores and concentrates and of waste and scrap were at low levels in both years. Other American countries were the most important supplier countries. The picture for metal exports is similar to that of the EU. Export shares of ores and concentrates grew from 3 to 8% during the period under review, and of scrap and waste from 11 to 17%. For both product groups, Asian countries became very important as destinations. The export share of products decreased significantly from 80 to 69%. Overall, Canada and Mexico are the most important trading partners for the US (see Appendix, Fig. 8).

## Discussion

The main results of the paper are discussed and grouped into two sections: (1) China is the global centre of metal production, consumption and trade; and (2) The EU and the US have lost shares in global metal production, consumption and trade.

1. China is the global centre in metal production, consumption and trade

China has become a major centre of industrial production in recent years. The main drivers of this development were favourable production conditions, good integration into global value chains, strategic partnerships and export-oriented industrialisation controlled by state leadership. For many foreign companies, China is also attractive because of its size as a labour and consumer market. In addition, joining the World Trade Organisation (WTO) in 2002 has been another important reason for its high growth rates (Schüler-Zhou et al. 2020).

The economic and industrial upswing has led to a sharp increase in the demand for raw materials. Considering material intensities, i.e. the ratio of consumed materials to GDP, China has recorded extremely high levels for base metals and steel for at least 20 years. In 2018, for instance, the Chinese economy consumed 77 tonnes of steel, 3.3 tonnes of aluminium, 1.2 tonnes of copper and 0.6 tonnes of zinc to generate a GDP of US$ 1 million (in constant 2010 US$). By comparison, the global economy consumed just 21 tonnes of steel, 0.8 tonnes of aluminium, 0.3 tonnes of copper and 0.2 tonnes of zinc for a GDP of US$ 1 million, with the EU and the US even below these levels for each metal (World Bank 2020, BGR 2021). A look at the most important economies worldwide and their material intensities for base metals and steel shows that currently, only Vietnam reaches similar levels of material intensities as China for some metals. In the past 60 years, only South Korea, Taiwan and Japan (and Poland for some metals) have recorded similar levels as China (BGR 2021).

**Fig. 3:**
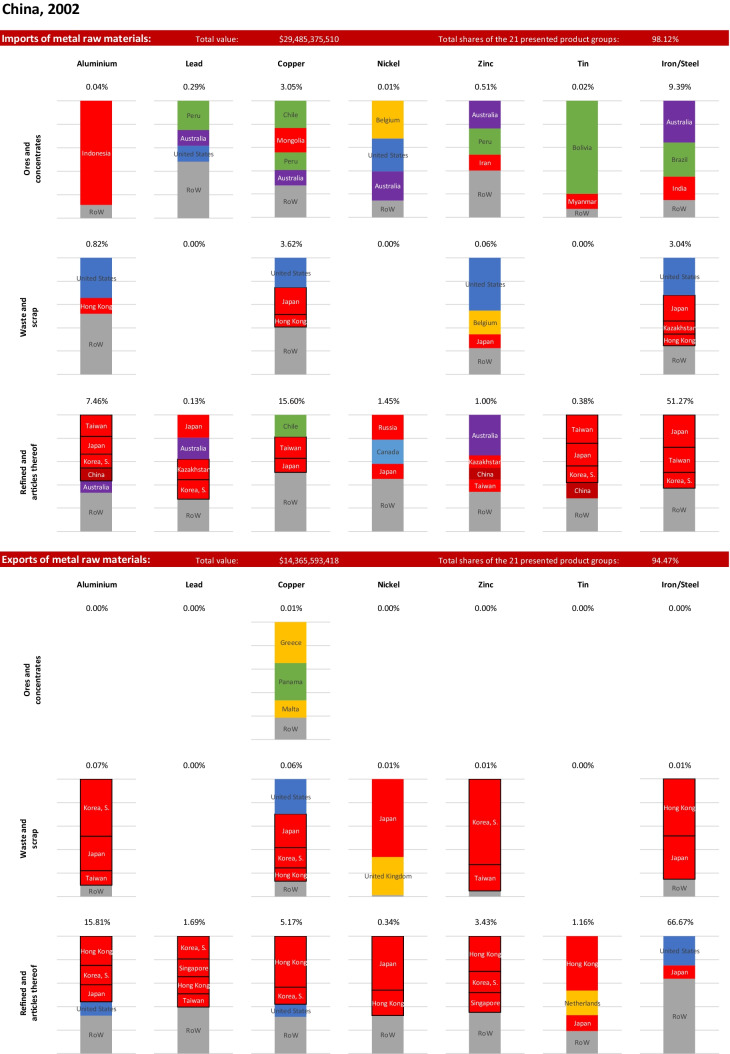
Distribution of trade in metal raw materials for China in 2002

**Fig. 4:**
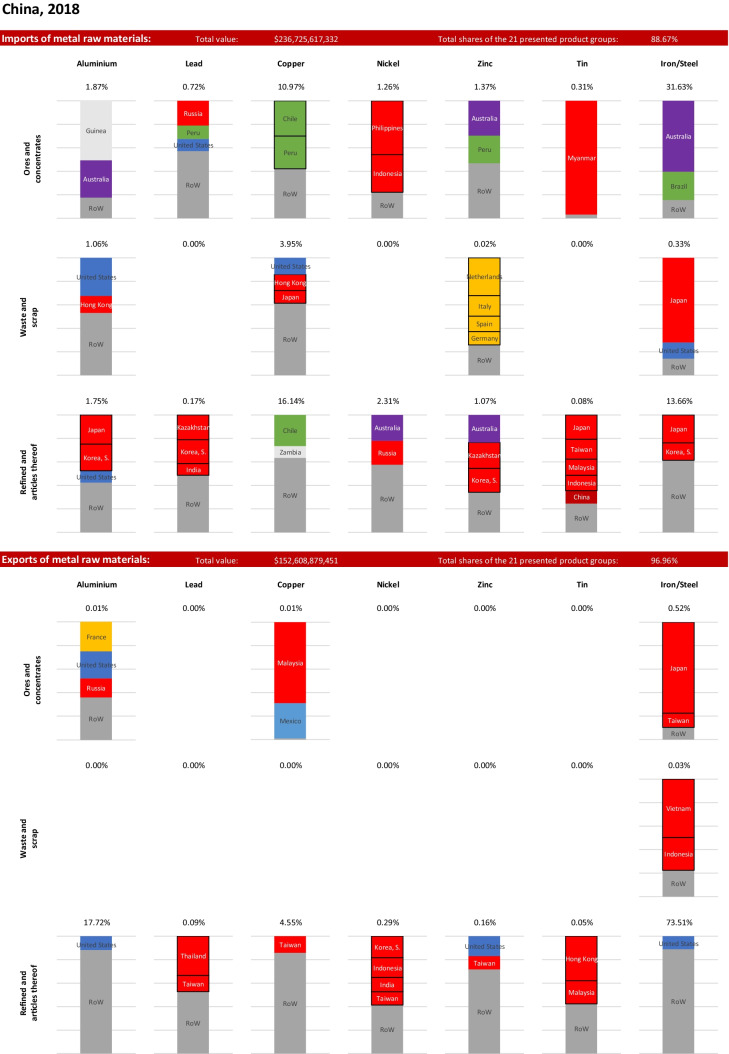
Distribution of trade in metal raw materials for China in 2018

Regarding foreign trade in goods from all product groups, China is the second largest importer country after the US,^2^ the largest exporter, and the largest net exporter (Comtrade 2020, in value)—and can therefore claim the title of world export champion. Considering foreign trade in metals, it is the biggest importer, exporter and net importer of metal raw materials in the world (GTA 2020, in value). China’s trade relationships has a far more global focus than it did at the beginning of the century: In 2002, Japan, Taiwan and South Korea were its most important trading partners for metal raw materials, in 2018, Australia had become its most important trading partner, followed by the US, Chile and Brazil. In 2002, trade in metallic raw materials with its direct neighbours accounted for 50% of total metal trade; in 2018, this value had fallen to 25% (BGR 2021, see in addition Figs. 3 and 4 in the Appendix).

With the proclamation of the Belt and Road Initiative (BRI) in 2013, its membership in the Regional Comprehensive Economic Partnership (RCEP) since 2020, and other measures, China, aims to secure its strong position in trade in the long term. Via BRI, China invests in infrastructure and transportation worldwide. Improving its conditions for trade by enhancing the transportation infrastructure especially in supplier countries but also in its markets is surely one of the many objectives China aims to pursue with the initiative (Chinese State Council 2015). Located in East Asia and Oceania, RCEP is the largest free trade zone in the world. Its 15 members are China, South Korea, Australia, New Zealand and the ASEAN countries Brunei, Indonesia, Cambodia, Laos, Malaysia, Myanmar, Philippines, Singapore, Thailand and Vietnam. These countries account for 30% of the world’s population, 30% of global GDP and 40% of global industrial production (World Bank 2020). They have agreed to reduce more and more trade restrictions and tariffs over time. This could make it easier especially for the biggest player in the region—namely China—to outsource the production of industrial pre-products and climb the value chain. RCEP can be expected to have trade-creating effects in the region (Flach et al. 2021).

Finally, some authors try to identify countries that could reach a similar dominant position on the metal markets as China in the predictable future. Stürmer (2012), Perger (2018) and Humphreys (2018) have analysed demand impulses in the metal markets of industrialising countries. Stürmer (2012) focused on the BRIC countries (Brazil, Russia, India and China), comparing their development with that of industrialised countries and analysing the consumption of aluminium, copper, crude steel, zinc and tin. According to this study, there are major differences between the individual BRIC countries. While China has undergone industrialisation processes over a period of 40 years, Brazil, India and Russia have been repeatedly set back by periods of stagnation. They are therefore unlikely to play a significant role in global commodity demand in the foreseeable future. Perger (2018) confirms the result with updated datasets that especially Brazil and Russia but also India are not expected to reach a dominant position on the demand side of the metal markets in the predictable future. Humphreys (2018) analyses in particular the booming nations of Southeast Asia, seeking to identify the next country that could play a similarly dominant role on the demand side of metal markets after China. While the author sees great development potential for the region, he does not expect any of the countries to be in a comparable position to China for global commodity demand in the foreseeable future. Although India and Indonesia are the countries expected to have the greatest impact, “there is no new China”.

(2) The EU and the US have lost shares in global metal production, consumption and trade.

Industrial production is still at a high level in both the EU and the US, growing by around half a percent per year. But both lost global share between 2002 and 2018, the EU’s declining from 25 to 18% and the US’ from 19 to 15% (see Fig. 1). Their losses in share of global metal consumption were even greater, declining from 26 to 14% for the EU and from 16 to 8% for the US (see Table 1). This gives an indication that their industrial production since 2002 has shifted away from the raw materials sector and towards the end of the value chains.

As a result, the EU and the US need to import pre-products and half-finished products on a larger scale (than China) while finishing takes centre stage. Imports of refined products and articles thereof accounted for 76% of metal imports to the EU and 91% to the US (see Figs. 6 and 8 in the Appendix). Although this might cut production costs, it leads to greater dependency on suppliers and supply chains. Recently, pandemic-induced lockdowns, as well as delivery delays and bottlenecks, have shown the disadvantages of globalised supply chains (Shih 2020). On the export side, refined products and articles thereof also play a dominant role for both the EU and the US, accounting for 83% of metal exports from the EU and 69% from the US (see Figs. 6 and 8 in the Appendix).

**Fig. 5:**
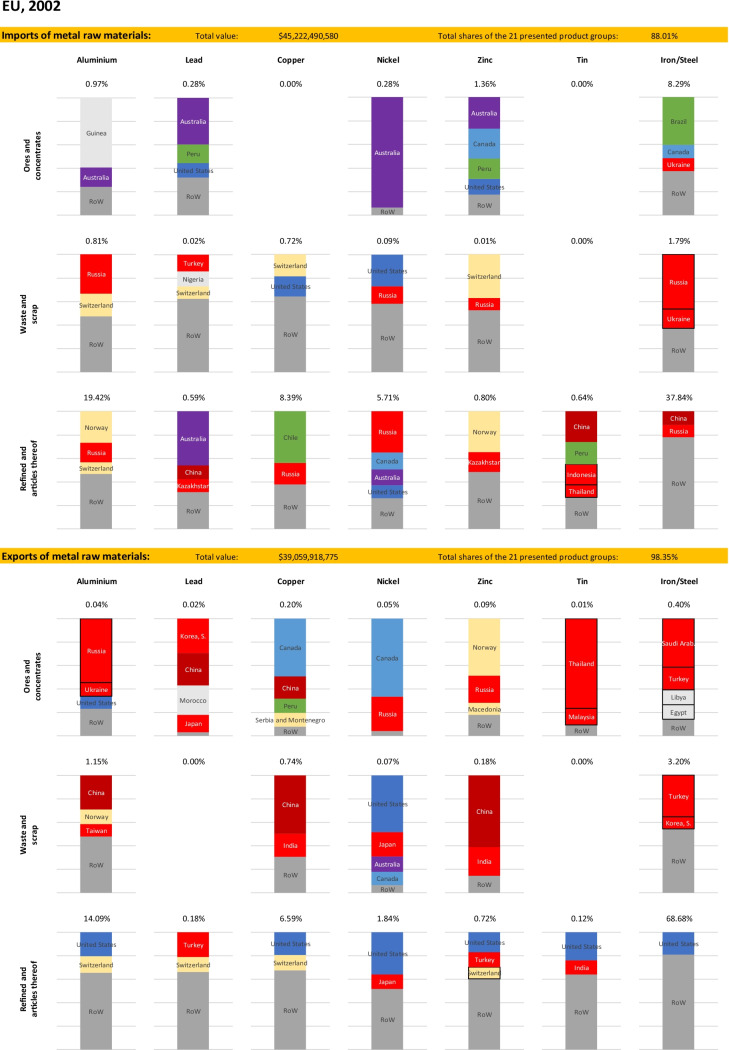
Distribution of trade in metal raw materials for the EU in 2002

**Fig. 6:**
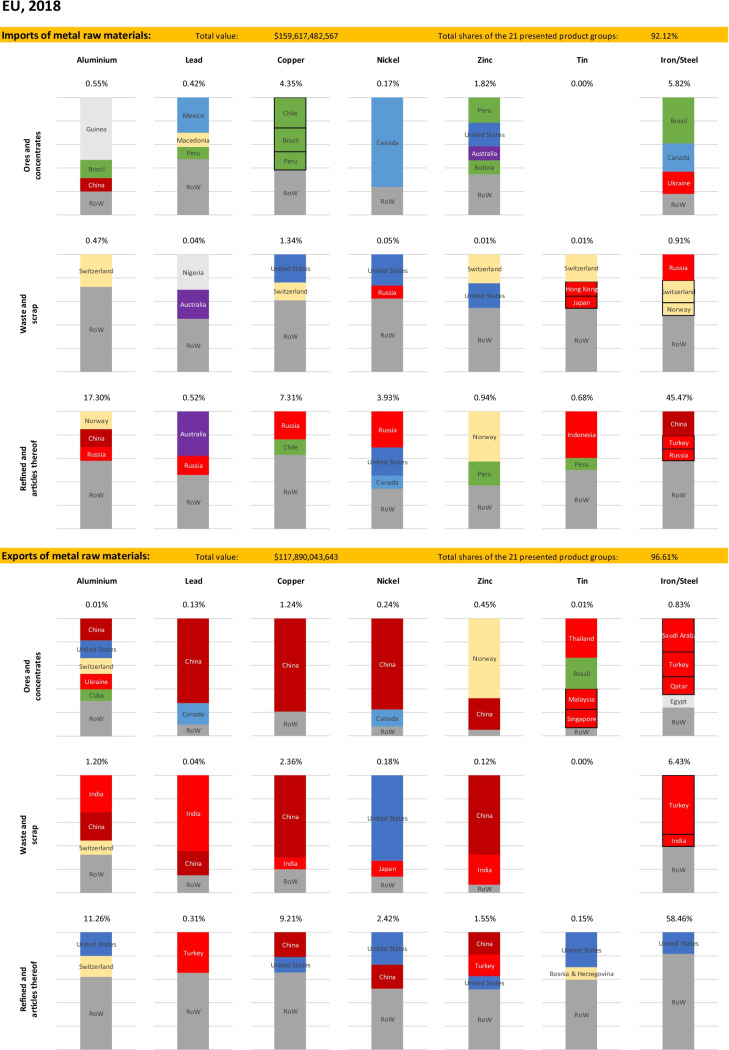
Distribution of trade in metal raw materials for the EU in 2018

**Fig. 7:**
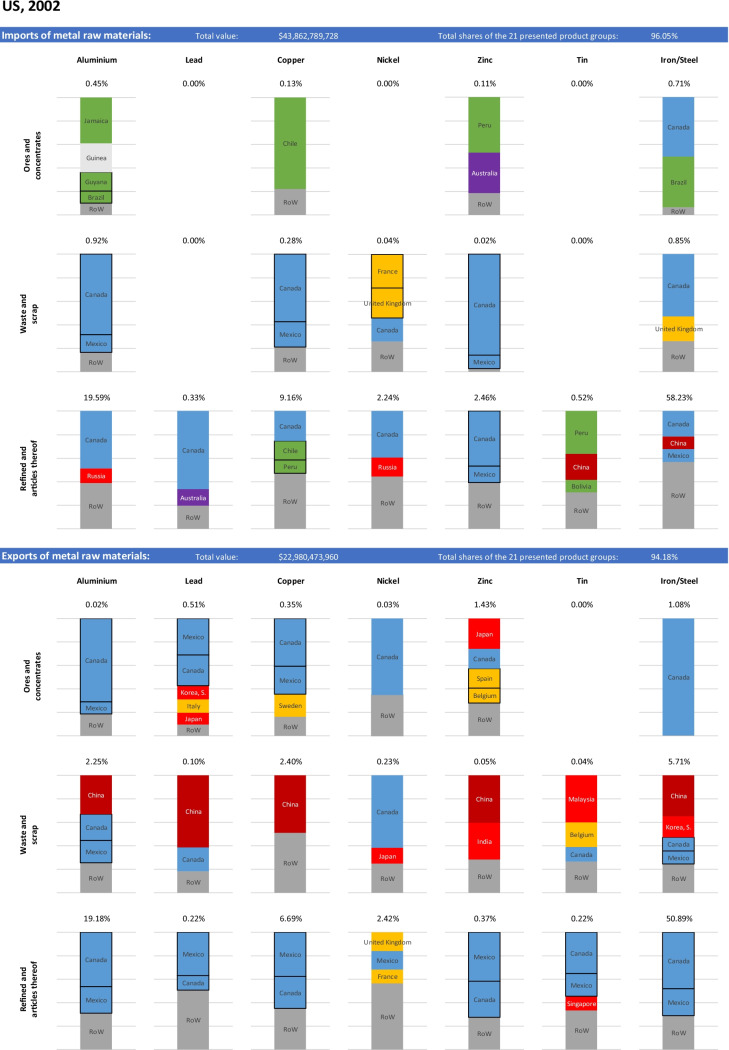
Distribution of trade in metal raw materials for the US in 2002

**Fig. 8:**
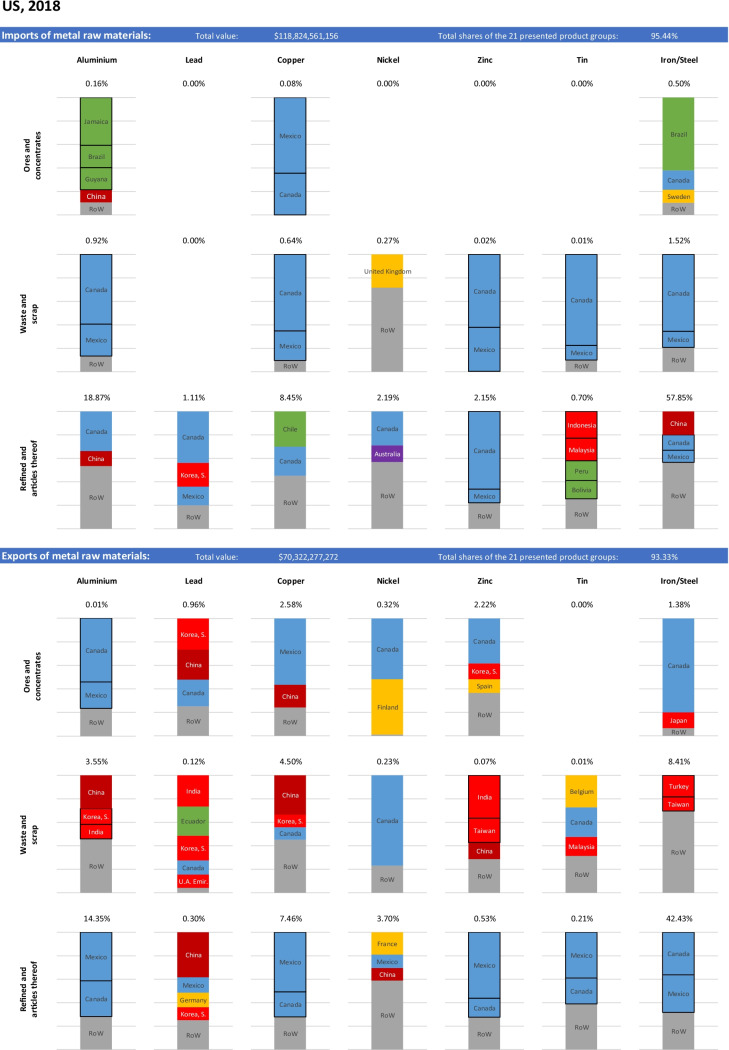
Distribution of trade in metal raw materials for the US in 2018

While the EU and the US export significant amounts of metal in the form of ores and concentrates or waste and scrap, China does not. In 2018, EU metal exports included 3% of ores and concentrates and 10% of waste and scrap, and US metal exports 8% of ores and concentrates and 17% of waste and scrap. All these shares increased from 2002 to 2018, while the shares of refined products and articles thereof declined (see Figs. 5 and 7 in the Appendix). In 2018, the EU exported ores and concentrates and waste and scrap mainly to China, India and Turkey; the US to Canada, China, India, Turkey, South Korea and Taiwan (see Fig. 6 and 8 in the Appendix). This shows the potential for reducing the dependence of the EU and the US on metal imports if the processing of metals was to be located more within the economic areas again.

## Conclusion and outlook

China has now overtaken the EU and the US in industrial production. To achieve this, the PRC has built up large mining capacities and enormous capacities for the refined production and refined consumption of metal raw materials. In 2018, China accounted for an average of 44.5% of global refined output of aluminium, lead, iron/steel, copper, nickel, zinc and tin, and consumed on average 49% of global refined products. The EU and the US consumed on average only 13.8% and 8% respectively of the refined products listed, and their average shares in global refined production were even lower. Absolute raw material consumption in China multiplied from 2002 to 2018, remained relatively constant in the EU and actually decreased slightly in the US. Overall, industrial production in China is more closely related to the raw materials sector, while in the EU and the US, it is located mainly in the higher processing stages and higher-value products. All three economic powers rely heavily on imports of raw materials. China’s significantly increased demand for raw materials has also turned it into the largest trader of metal raw materials. Moreover, China’s commodity trade has become much more internationalised over the years. In 2018, China no longer traded primarily with its Asian neighbours, but around the globe, increasingly displacing the EU as the most important trading partner for many countries. The US have long been active as a major trading partner of metal raw materials, especially in North America.

China’s dominant position in the base metals commodity markets gives it great market power. From this position, it is possible for the PRC to significantly influence prices and control supply chains in the raw materials sector. In the COVID-19 pandemic, its importance on global metal markets has grown according to preliminary data even more.

## Figures and Tables

**Table 3:**
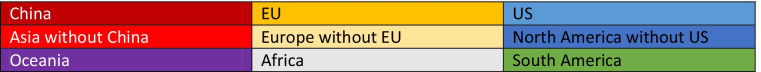
Detailed distribution of metal trade

## Appendix

Table 2

**Table 2:** Analysed Harmonised System codes (HS codes)

Aluminium ores and concentrates	2606
Aluminium waste and scrap	7602
Aluminium refined and articles thereof	76 without 7602
Lead ores and concentrates	2607
Lead waste and scrap	7802
Lead refined and articles thereof	78 without 7802
Iron ores and concentrates	2601
Iron/Steel waste and scrap	7204
Iron/Steel refined and articles thereof	72 and 73 without 7204
Copper ores and concentrates	2603
Copper waste and scrap	7404
Copper refined and articles thereof	74 without 7404
Nickel ores and concentrates	2604
Nickel waste and scrap	7503
Nickel refined and articles thereof	75 without 7503
Zinc ores and concentrates	2608
Zinc waste and scrap	7902
Zinc refined and articles thereof	79 without 7902
Tin ores and concentrates	2609
Tin waste and scrap	8002
Tin refined and articles thereof	80 without 8002
Non-base metals	81 and 26 without base metal ores and conc
Metals	26, 72, 73, 74, 75, 76, 78, 79, 80, 81

Table 3

Figure 3

Figure 4

Figure 5

Figure 6

Figure 7

Figure 8

## Footnotes

1 The commodity trade groups considered as defined within the Harmonised System (HS) are found in Appendix Table 2.

2 The EU also ranks above China, but it is not a single country.
